# Acceptance of Public Health Measures by Air Travelers, Switzerland

**DOI:** 10.3201/eid1505.080933

**Published:** 2009-05

**Authors:** Nicole Senpinar-Brunner, Tobias Eckert, Kaspar Wyss

**Affiliations:** Author affiliations: Swiss Tropical Institute, Basel, Switzerland (N. Senpinar-Brunner, T. Eckert, K. Wyss); and Federal Office of Public Health, Bern, Switzerland (T. Eckert)

**Keywords:** Bioterrorism and preparedness, disease outbreaks, communicable disease control, quarantine, patient isolation, information dissemination, travel, transportation, public health practice, letter

**To the Editor:** Infectious diseases can spread rapidly by air travel, as did severe acute respiratory syndrome (SARS) in 2003. Public health measures at airports might protect passengers and employees from such diseases and delay spread into the general population. The SARS epidemic was contained largely through traditional public health interventions (*1*,*2*). These interventions included recommendations to postpone nonessential travel, provide public health information and face masks, screen passengers at entry or exit by questionnaire, measure ear temperatures, provide medical examinations, isolate case-patients, and quarantine contacts.

For a future influenza pandemic, the World Health Organization does not encourage entry screening for any pandemic phase but leaves this decision for screening to each country (*3*). Switzerland currently considers entry screening, albeit not by infrared thermal scanning (*4*,*5*). Many travelers to developing countries do not obtain health information or use preventive measures (*6*). However, to be effective, public health measures must be communicated to and accepted by travelers. Current knowledge on acceptance of public health measures by air passengers is limited. Compliance with quarantine measures seems to depend on consistency of policies and credibility of public health messages (*7*).

To investigate passenger knowledge, communication preferences, and acceptance of public health measures for a hypothetical respiratory disease pandemic, we conducted a cross-sectional survey among passengers departing from EuroAirport Basel-Mulhouse-Freiburg (Haut-Rhin, France) to European destinations, and from Zurich Airport (Kloten, Switzerland) to Asian or North American destinations during the summer of 2007. Data were collected by a pretested, self-administered, 23-item questionnaire (in English, French, and German), which was distributed to all adult passengers in the departure waiting areas. Information was analyzed by basic statistical methods, χ^2^ and *t* tests, and logistic regression adjusting for sex, age group, airport, residence region, solitary traveling, and business travel.

A total of 2,633 passengers were approached and asked to participate. The response rate was 71%. Most passengers who refused to participate did so because of language difficulties. After we excluded passengers <18 years of age, data for 1,880 participants (1,081 at EuroAirport and 799 at Zurich Airport) were analyzed. Mean (SD) age was 39.8 (14.7) years; 54% were female, 58% had a university degree, 97% were currently feeling healthy, 30% were traveling alone, 37% were traveling for business reasons, 30% were residents of Switzerland, 42% were residents of other European countries, and 15% were residents of the Americas.

Passengers were asked about acceptance of public health measures in a hypothetical severe respiratory disease pandemic. Results are shown in the Appendix Figure. A total of 71.6% would cancel their trip if postponement of nonessential travel was recommended, 93.7% would wear face masks, 93.2% would fill out a health questionnaire, and 89.1% would accept having their ear temperature measured on arrival. If fever were detected, 88.1% would undergo a short physical examination. If persons were diagnosed with a disease and were receiving treatment, 92.3% would accept isolation for 7 days. If feeling healthy but were seated next to someone with a cough on the airplane, 69.2% would accept 7-day quarantine at home (residents of Switzerland) or hotel (travelers to Switzerland) and would monitor their health. Fewer persons from the Asia-Pacific region would accept these requirements. Male passengers and all passengers >30 years of age indicated they would be more compliant than other passengers with nearly all measures. However, many female travelers explained that they would not consider traveling during a pandemic.

There were no differences in questionnaire responses between the 2 airports. Other questions concerned information status and seeking. In a pandemic, 93.5% of passengers would acquire information before departure about the situation and preventive measures; 67% would consult the Internet, 59% their family doctor, 49% the media, 37% health authorities, 25% their travel agent, 23% travel medicine centers, 20% the airport, and 17% friends and relatives. For their current trip, 22.4% sought pretravel advice on infectious diseases. This seeking of advice was more frequent in those departing to overseas destinations from Zurich Airport. Information sources were the family doctor (38%), the Internet (34%), the media (26%), friends and relatives (22%), a travel agency (19%), health authorities (11%), travel medicine centers (10%), and the airport (7%). A total of 17.4% noticed the official posters regarding avian influenza.

Because the study was conducted when no major international disease outbreaks were occurring, passengers answered hypothetical questions about an imaginary future pandemic. Therefore, attitudes and behavior might be difficult to predict from these results and would depend on perceived severity of the pandemic disease. Similar surveys among the general population showed comparable results. During an influenza pandemic, 75% of Europeans would avoid public transportation (*8*). In the United States, 86% would stay at home in quarantine and 94% would stay in isolation (*9*). A survey in Hong Kong Special Administrative Region, People’s Republic of China reported 74% would wear face masks in public, 87% would make declarations at border crossings, and 88% would comply with quarantine policies (*10*).

## Supplementary Material

Appendix Figure Acceptance of potential public health measures and recommendations for a hypothetical pandemic respiratory disease among 1,055 air passengers at EuroAirport (EA) in Haut-Rhin, France, and 782 air passengers at Zurich Airport (ZK) in Kloten, Switzerland.
