# Supplementary figures and images for: Acceptance of Public Health Measures by Air Travelers, Switzerland
Source: Emerg Infect Dis. 2009 May;15(5):831–2. doi: 10.3201/eid1505.080933 (PMC2687032; doi:10.3201/eid1505.080933)

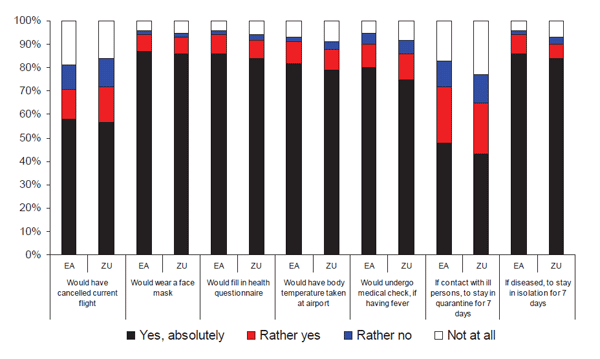

Supplement: Appendix Figure — Acceptance of potential public health measures and recommendations for a hypothetical pandemic respiratory disease among 1,055 air passengers at EuroAirport (EA) in Haut-Rhin, France, and 782 air passengers at Zurich Airport (ZK) in Kloten, Switzerland. [file 08-0933_app-s1.gif]
